# Impact of tumour size on metastasis and survival in patients with pancreatic neuroendocrine tumours (PNETs): A population based study

**DOI:** 10.7150/jca.27779

**Published:** 2019-10-18

**Authors:** Yangyang Liu, Shufang Ye, Yabi Zhu, Xingkang He, Jie Pan, Shujie Chen, Bin Ye, Liangjing Wang

**Affiliations:** 1 Department of Gastroenterology, Sixth Affiliated Hospital of Wenzhou Medical University, Lishui People's Hospital, Lishui, Zhejiang Provine, China; 2 Institution of Gastroenterology, Zhejiang University, Hangzhou, Zhejiang Province, China; 3 Department of Gastroenterology, Sir Run Run Shaw Hospital, Zhejiang University, Hangzhou, Zhejiang Province, China Department of Gastroenterology, Zhejiang University Lishui Hospital,; 4 Department of Endocrinology and metabolism, Second Affiliated Hospital of Zhejiang University School of Medicine, Hangzhou, Zhejiang Province, China; 5 Department of Gastroenterology, Lishui Municipal Central Hospital, Lishui, Zhejiang Province, China; 6 Department of Gastroenterology, Second Affiliated Hospital of Zhejiang University School of Medicine, Hangzhou, Zhejiang Province, China

**Keywords:** tumour size, pancreatic neuroendocrine tumours, PNETs, metastasis, survival

## Abstract

**Background:** The relationship between tumour size and metastasis rate is poorly recognized in patients with pancreatic neuroendocrine tumours (PNETs). The impact of tumour size on prognosis was controversial in previous investigations.

**Methods:** PNETs cases diagnosed from 1988 to 2013 were retrieved from the Surveillance, Epidemiology, and End Results (SEER) database. Clinicopathologic features were retrospectively analyzed. Survival was calculated by the Kaplan-Meier method. Multivariable Cox regression models with hazard ratios (HRs) were constructed to analyze survival outcomes and risk factors. Cubic spline analysis was used to assess relationship between tumor size and probability of metastasis.

**Results:** A total of 5424 patients were identified, 1226 (22.6%) with tumour size of 20mm or less. The probability of metastasis increased in a non-linear fashion with increasing tumour size. Univariate analysis showed that tumour size was significantly correlated with survival (*P*<0.001), no matter surgery was performed or not. However, subgroup analysis suggested this association to be linear for patients with localized and regional tumours (*P*<0.001), but stochastic in patients with distant stages (*P*=0.703). On multivariate analysis, tumour size was an indicator for metastasis (HR=1.010, 95%CI: 1.008-1.013, *P*<0.001) and size≤20mm was an independent prognostic factor for good survival. For tumours≤20mm, surgical treatment was associated with significantly improved survival (*P*<0.001).

**Conclusions:** Tumour size affects the probability of metastasis. Its prognostic impact on survival is restricted to patients with localized and regional disease. For patients with tumour size ≤20mm, surgical treatment should be considered preferably.

## Introduction

Neuroendocrine tumours (NET) consist of a diverse group of neoplasms that derive from diffuse neuroendocrine cells throughout the body^1^. Commonly found in gastrointestinal (GI) duct and lung, they also arise in the pancreas. Pancreatic neuroendocrine tumours (PNETs) are relatively rare, accounting for approximately 7% of all neuroendocrine tumours, and <3% of overall pancreatic tumours^2^,^3^. According to data from the National Cancer Institute Surveillance, Epidemiology, and End Result (SEER) registry, the overall incidence is 0.43 cases per 100,00 inhabitants, which has more than doubled in the past 20-30 years^2^,^4^. This is partially due to the increased physician awareness, improvements in recognition of neuroendocrine histology and advances of diagnostic techniques^1^.

PNETs exhibit heterogeneous biologic behavior, ranging from indolent to aggressive based upon specific histology which may include the elaboration of active gastrointestinal hormones^1^. Depending on the presence or absence of clinical syndromes resulting from an inapproapriate hormone secretion, PNETs are divided into 'functional' and 'non-functional' subgroups. About 60-90% of patients are non-functional, largely asymptomatic. In contrast, functional PNETs have variable clinical presentations caused by different hormones, such as insulin, gastrin, vasoactive intestinal peptide(VIP), glucagon, somatostatin and secroton^5^.

Given the heterogenous nature of PNETs, efforts to identify reliable prognostic features have been a challenge. Tumour size is one of these essential values. The proposed American Joint Committee on Cancer (AJCC) and European Neuroendocrine Tumours Society (ENETS) classifications have defined disease stage based on size of primary tumour^6^,^7^. However, validation of these systems has been conflicting, and tumour size has not been shown to be significantly predictive of survival in several studies^8^,^9^,^10^. Even more, Kuo and his colleagues reported a relatively high rate of metastasis in small tumours^11^. Nonetheless, the metastatic rate of PNETs of all sizes has not been reported before.

Surgical resection is the only potentially curative therapy for PNETs, and palliative surgery is also an accepted course of action in cases of liver metastatic disease^12^,^13^. However, for tumours no larger than 20mm, surgical treatment remains to be controversial. As stated in the ENETS guidelines^14^ and some scientific literatures, conservative approach was recommended to be safe^15^,^16^,^17^, while some others reported that long-term outcome of resected patients were better than those with non-operative treatment^18^.

Most of the above studies have been based on data from single-center institutions. The aim of the present study is to investigate the relationship between primary tumour size and metastatic rates and survival in patients with PNETs, and to explore the impact of surgical intervention on tumours ≤20mm using a population-based registry.

## Materials and Methods

### Data source

The SEER registry database, sponsored by the National Cancer Institute was used for this study. It includes over three million cases from 18 geographic sites among 14 states from the USA, representing approximately 30% of the US population. The SEER database records patient demographics (e.g. age and sex), primary tumour characteristics (e.g. size, extent and grade), nodal staging (number of nodes examined and number of involved nodes), primary operation performed, vital status and survival. The July 2016 update was used for this study, providing information from 1973 to 2013^19^. Quality control is an important component of the SEER program, and the current standard for accuracy of the data in the registry is an error rate of less than 5%^19^. We have got permission to access the research data file using SEER*Stat version 8.3.2 and the reference number was 12907-Nov2015. This study was approved by the Institutional Review Board.

### Inclusion and Exclusion Criteria

As detailed tumour size was not recorded before 1988, our study group included all patients with PNETs registered in the SEER database between 1988 and 2013. Patients diagnosed after 2013 were excluded to ensure an adequate duration of follow-up. The specific inclusion criteria were as follows: 1) the years of diagnosis ranged from 1988 to 2013. 2) the International Classification of Disease for Oncology (3rd edition) site record was limited to the following items: islet cell carcinoma (8150), insulinoma (8151), glucagonoma (8152), gastrinoma (8153), mixed islet-cell/exocrine adenocarcinoma (8154), vipoma (8155), somatostatinoma (8156), carcinoid (8240), enterochromaffin cell carcinoid (8241), enterochromaffin-like cell tumors (8242), goblet cell carcinoid (8243), composite carcinoid (8244), adenocarcinoid (8245), neuroendocrine carcinoma (8246), and atypical carcinoid (8249). 3) site record ICD-O-3 was limited to the pancreas. 4) patients with histologically confirmed disease and tumour of all grades were included in the analysis. 5) Tumour size was measured as the maximum length of the tumour based on the pathological, operative or radiological report, in this order of priority. The exclusion criteria were as follows: 1) patients with a lack of documentation of their race, marital status, or age at diagnosis were excluded; 2) patients with incomplete follow-up were removed.

The following factors were retrieved from the SEER database: the year and age at diagnosis, sex, race, tumour stage, site record, histological grade, surgical resection, regional nodes positive, tumour size, metastasis, survival months, vital status.

In order to ensure a coherent cancer staging classification across the study period, the “SEER historical stage” was used, which provides a consistent definition over time. It is a coding schema with 3 clinically relevant categories: localized, regional, or distant disease. Although the AJCC staging system was more widely used in clinical practice, it was not accessible for many of the annual data sets analyzed.

### Statistical analysis

Incidence rates per 100,000 were calculated using SEER*Stat. Univariate analyses comparing patient demographics and tumor characteristics were performed. Survival curves were calculated using the Kaplan-Meier method, and the log-rank test was carried out to evaluate the survival differences between groups. Risk factors with a *P* value<0.1 in the univariable analysis were entered into the multivariable analysis. The Cox proportional hazards model was built to calculate adjusted hazard ratios (HRs) along with 95% confidence intervals (CIs), which were used to assess the strength of the individual variables. To graphically demonstrate a relationship between tumour size and probability of metastasis, cubic spline analysis was conducted—which makes no assumption about the relationship between parameters and is entirely data-driven. Logistic regression was used to assess the prognostic value of tumour size for the presence of metastasis. Data was analyzed using IBM SPSS Statistics version 23.0 for Microsoft (IBM Corp. Armok, NY, USA). When the two-sided* P*-value was less than 0.05, the difference was considered statistically significant.

## Results

### Demographics and tumour characteristics

**Figure 1:**
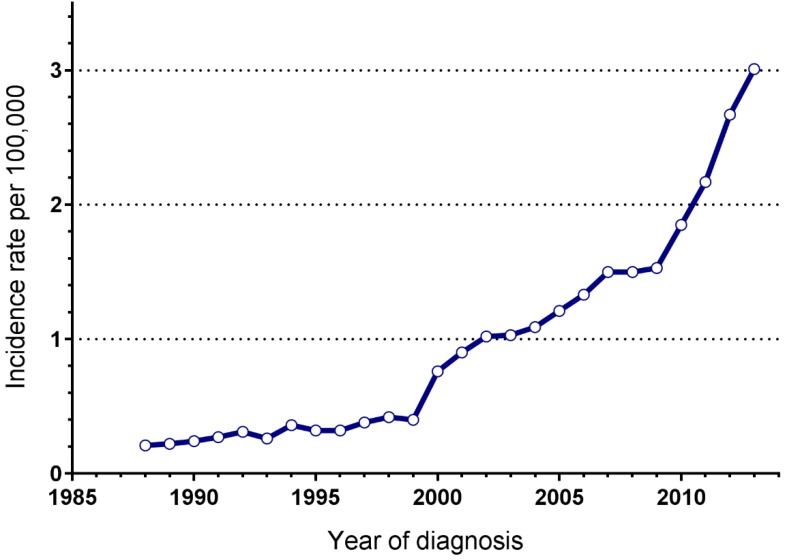
Annual incidence of PNETs, Surveillance, Epidemiology, and End Results registry 1988 to 2013.

In total, 7074 patients with PNETs were registered in the SEER database during the 25 years study period. Some 1650 patients did not fulfill the inclusion criteria, not available for detailed information on follow-up or tumour size and were excluded. Finally, 5424 eligible patients were included in the study group and were used for further calculations. They comprised about 77% (5424/7074) of the total number of PNETs. The annual incidence rate of this disease was significantly increasing over time, from 0.21/100,000 in 1988 to 3.01/100,000 in 2013 (**Figure 1**). Patients and detailed tumour characteristics are summarized in **Table 1**. Briefly, most patients were white (80.5%), with a male predominance (male: female=1.24) and a median age of 61 years old (interqurtile range, IQR: 50-71). A total of 4279 patients (78.9%) presented with a functioning tumour, while 1145 (21.1%) with non-functioning. Tumours were commonly located in pancreatic head (33.5%), tail (31%), body (13.8%). Most patients had metastatic tumours (45%) at the time of diagnosis, with 31% had localized disease, 22.4% had regional disease, and in 1.6% of patients the disease stage was unknown. The median tumour diameter was 46.2mm, 22.6% of the tumours had a diameter of 20mm or less, and 44.1% of the tumours were larger than 40mm.

### Survival and prognostic factors

The median overall survival (OS) for all cases was 21 months (range 0-485months), and we observed that young age (≤60 years), race of unknown, female sex, functional tumours, location of others, low tumour grade, localized tumour stage, surgery, negative regional lymph node and small tumour size showed a significant relationship with increasing overall survival based on univariate analysis. Next, we carried out a multivariate analysis, all factors that were associated with survival were included in the Cox-regression model. It showed that the age at diagnosis, tumour grade, tumour stage, surgical resection and tumour size were independent prognostic factors. The tumour stage was the most influential predictor, with the highest HR. However, race, gender, primary site and regional lymph node status were not predictive of outcome (**Table 2**).

Subgroup analysis was undertaken to determine whether the prognostic impact of tumour size was consistent across stage and treatment categories.

### Survival according to tumour size and stage

**Figure 2:**
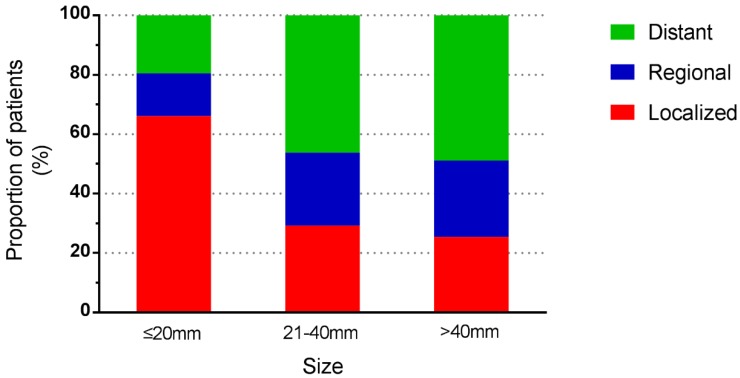
Stage distribution according to tumour size.

**Figure 3:**
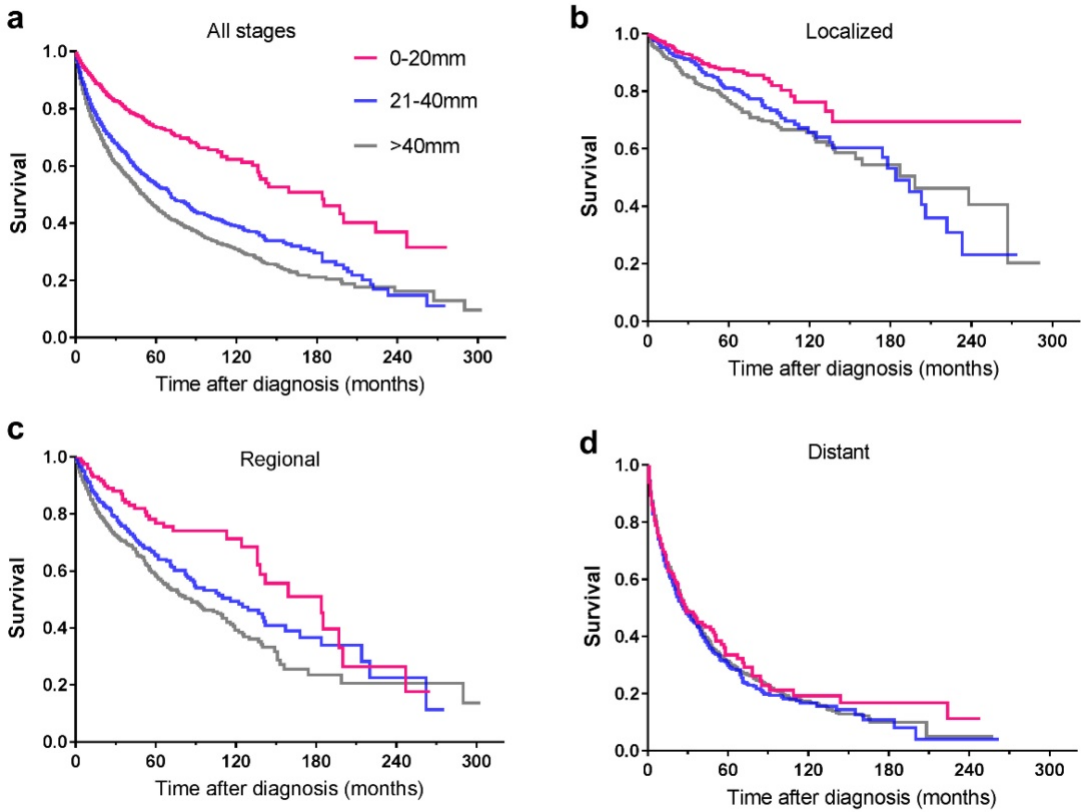
Kaplan-Meier survival curves illustrating survival by tumour size (mm) and stage of disease: a. all stages; b. localized; c. regional; d. distant

Among the included 5424 patients, 90 patients were unstaged. Therefore, a total of 5334 patients were enrolled for further study. Tumour stage distribution according to tumour size is shown in **Figure 2**. When the tumour size was ≤20mm, the rate of distant metastasis was 19.5%, increasing to 58.9% when the tumour size was larger than 40mm. The 5-year survival rate for the whole study group was 51.5%. For tumours ≤20mm, the 5-year survival rate was 71.8%, and the rate decreased to 43.8% in patients with tumours>40mm. For patients of localized and regional disease stages, the survival rates decreased with increasing tumour size, while for patients of distant disease stage, tumour size no longer affected the survival rates dramatically (**Table 3**). Among 1681 patients with local disease only, the 5-year survival rate was 81.4%. It was 87.1% for patients with tumours of 20mm or less in diameter, decreasing to 75% for those with tumours larger than 40mm. In patients with regional disease, survival was similar across tumour size categories. The relationship between tumour size and survival, stratified by stage, is shown in **Figure 3**.

### Survival in patients who underwent surgery

**Figure 4:**
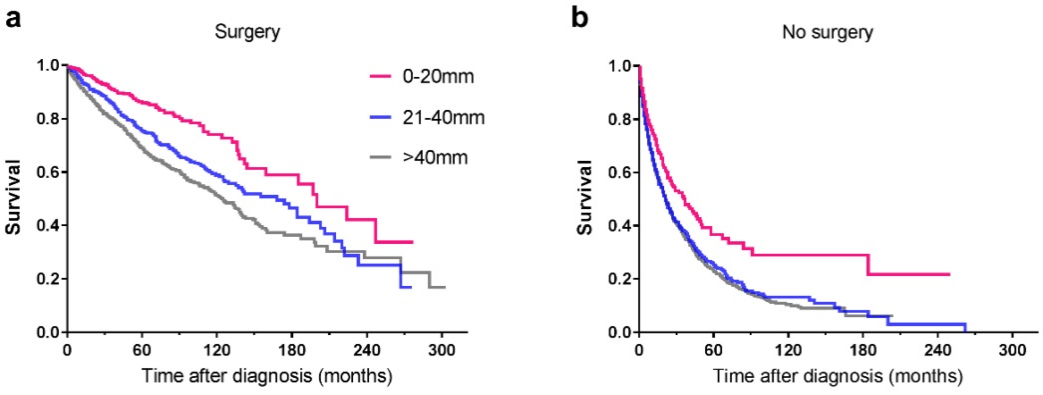
Kaplan-Meier curves of patients treated with (a) surgical resection and without (b) surgery, showing survival by tumour size.

**Figure 5:**
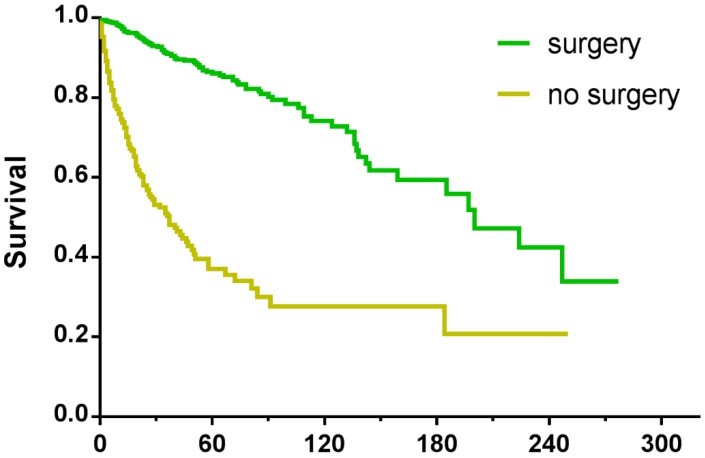
Kaplan-Meier survival curves of patients with tumour size≤20mm, treated with or without surgery.

Among the 5424 patients, information regarding surgery was unknown in 15 cases. As a result, the remaining 5409 patients were enrolled for further study. Some 3041 patients underwent surgical treatment, the 5-year survival rate of whom was 74.7% compared with 23.4% for patients who were not operated on. For patients with tumours ≤20mm, no matter surgery was performed or not, they had better survival than those with larger tumours (**Table 4, Figure 4**). In order to explore the significance of surgery on small PNETs, we performed a further study in patients with tumour size≤20mm. For those who underwent surgery, the 3-year and 5-year survival rates were 90.5% and 85.6% respectively, absolutely more favorable compared with 46.6% and 33.9% for those who did not receive operation (*P*<0.001) (**Figure 5**).

### Size and metastasis

**Figure 6:**
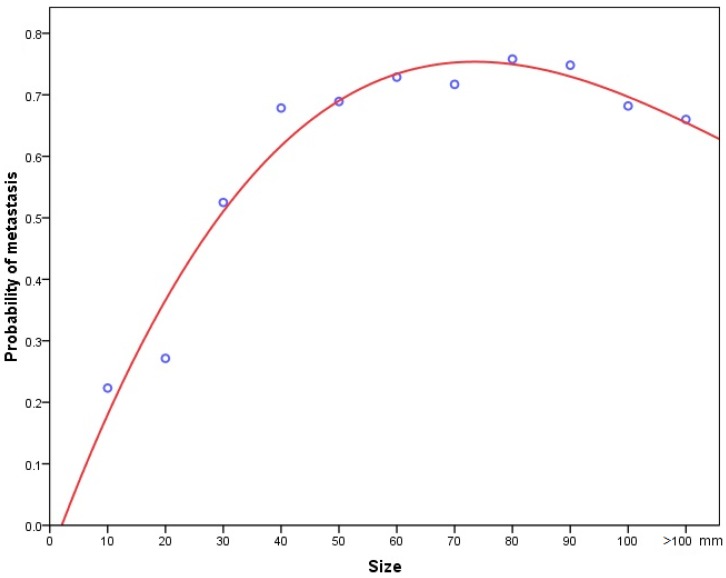
A cubic spline graph showing association between tumour size and the probability of metastasis.

When pathologically confirmed tumours were classified into 10mm size categories, there was a positive correlation between increasing tumour size and the probability of metastasis. Initially, it was analyzed with linear model, but we noted an obvious lack of fit. Then cubic spline analysis was used instead to generate a logistic curve, which was found to fit the data much better than the linear size (**Figure 6**). A multivariate logistic regression analysis revealed that a size increment of 1mm was an independent prognostic factor for metastasis (HR=1.017, 95% CI: 1.014-1.019, *P*<0.001).

### Characteristics of PNETs≤20mm

As shown in Table 2, we found that tumour size≤20mm was an indicator of good prognosis. In order to clarify the unique features of PNETs with size of 20mm or less, its main parameters were compared with those of larger tumours. Among the identififed 5424 cases, 1226 tumours were ≤20mm and 4198 were>20mm. Demographic, clinical, and pathologic characteristics were summarized in Table 5. Briefly, most patients with tumour size≤20mm were well differentiated (52.8%) and had localized disease (65.1%), which both indicated good survival.

## Discussion

The SEER program is an excellent tool for population analysis of malignancies, especially of rare diseases because of its data collection for over 30 years, extraordinary accuracy, and close approximation to the general US population^19^. Therefore, we conducted this study to elucidate some aspects of incidental trends, tumour characteristics and prognostic factors in patients with PNETs. Franko and his collegues have previously reported on PNETs using SEER data up to 2004^10^. However, they included only non-functional tumours with a smaller sample size and gave limited information. We herein provided an updated and more comprehensive evaluation of the incidence and prognosis of these uncommon tumours. The strength of our study is that the data include all patients diagnosed in a whole country over a 25-year period. This eliminates the risk of inclusion bias or referral bias.

The annual incidence of PNETs is nearly 3.01/100,000 in the population, and this has been increasing over time. Consistent with previous studies^8^,^10^, we found that the majority of patients were men, white, and non-functional. The populations in our study showed a similar prognosis, with a 5-year survival rate of 51.6%. Not surprisingly, we observed that age, pathological grade, tumour stage, surgical treatment and tumour size were independent prognostic factors in multivariate analysis. Higher grade, more advanced stage, surgical treatment, higher age at diagnosis and larger tumour size were the strongest predictors of worse survival. However, our analysis did not reveal any statistical differences in race, gender and tumour location, which is in keeping with the findings of other investigators^8^,^10^. Regarding the effect of functional status on survival, a majority of studies had reported a positive correlation. They found that patients with functional tumours had a more favorable prognosis than non-functional tumours^20^,^21^,^22^. However, in our study, there was no statistical significance of functionality on survival outcomes. This may be mostly attributed to the limitations of the SEER registry. The SEER registry did not provide data on the clinical presentation or laboratory values which were used to make the distinction between functional and non-functional tumours. We just used the histological codes to determine the functionality of PNETs, which relied on pathology reports supplied by the participating institutions, and this gave rise to a potential for misclassification of these tumours. Another important reason was that the SEER registry excluded PNETs considered to be benign, which consisted of a number of functional tumours and manifested a quite favorable prognosis.

Traditionally, it is recognized that the presence of lymph nodes has negative impact on survival. However, whether it is an prognostic factor has always been conflicting, as different investigations giving different results^14^ . A number of studies reported that lymph node status had important prognostic value^9^,^23^,^24^,^25^,^26^. However, other reports failed to detect a survival difference in those with lymph node diseases^27^,^28^,^29^. In the current study, we also found that lymph node involvement had no effect on survival. As this is an important management point because it has a direct influence on the type of and extent of surgical procedure that should be performed, more investigations, especially larger and prospective, are demanded.

Tumour size is a readily available parameter, can be accurately measured and has the great advantage of little discrepancy among observers. Its prognostic value in PNETs has been investigated extensively 8,9,10,30,31,32,33. Although the results beween different studies were inconsistent, tumour size was used as a criterion of staging in the AJCC and ENETS system^6^,^7^. In the present study, we demonstrated that tumour size was an independent prognostic factor for survival among patients with PNETs, on both univariate and multivariate analysis. More precisely than before, we revealed a stage-dependent relationship between tumour size and survival. The association between small tumour size and prolonged survival was confirmed in the subgroups of patients with localized and regional cancers. Once the cancer had disseminated, tumour size was no longer an important predictive factor for long-term survival. The possible reason may be that for distant disease, patients already had metastasis, thus the influence of tumour size on their prognosis was likely outweighed by the dismal outcomes associated with the pre-existing metastatic disease. Furthermore, we provided the first evidence that increasing tumour size was associated with a higher probability of metastasis in a nationwide registry, starting at 13% for tumours 10mm or less and attaining 52% for tumours greater than 90mm. A nonlinear relationship was noted, with a sickle shape. The possible explanation was the propensity and opportunity of individual cells in a tumour to metastasize. In smaller tumours, by contact with adjacent structures, the odds for cells to gain access to vascular or lymphatic channels increased, which consequently lead to dissemination. While, in larger tumours, central necrosis, due to the lack of a stable blood supply, may contributed greatly to the decreased proportion of metastatic rate, which account for the sickle shape that we observed.

Regarding PNETs≤20mm, some prior reports found that the proportion of these patients has nearly doubled over the last 22 years^11^. This increase was probably because of the frequent use of axial imaging and endoscopic ultrasound. For these patients, an aggressive surgical approach has long been the rule^34^,^35^. However, despite its increasing safety, surgical resection has recently been challenged because of its lack of proven effect on long-term survival^36^,^37^. Thus, the optimal treatment for these patients became controversial. Recently, for this subgroup of lesions, a wait-and-see policy has been advocated^38^. Nevertherless, we herein showed that patients with PNETs≤20mm who undergo surgery demonstrate a distinct survival benefit compared with their unresected peers. So we still recommended surgery as the preferred treatment for these patients.

Our present study had some limitations. First, it was retrospective and had the general weakness of this study design. Second, as a population-based registry, the SEER database could have coding errors inevitably, and it provided a large sample size at the expense of loss of clinical details. Third, in order to classify the tumour stage, the SEER historical stage A was used, which was different from the AJCC staging system and probably of less clinical relevance. However, these classifications were available for most individuals and keep consistent during the whole study period.

Despite the above limitations, our findings brought important clinical values. This large population-based study provided an up-to-date estimate of the incidence data as well as clinicopathological characteristics and survival analysis of PNETs. It suggested that tumour size correlated strongly with the rate of metastasis and was an independent prognostic factor of survival after adjusting for confounding variables. Surgical treatment was preferably recommended for PNETs, including patients with tumour size≤20mm.

## Figures and Tables

**Table 1:** Clinicopathological characteristics and univariate analysis of patients with pancreatic endocrine tumours in the SEER registry 1988-2013

Characteristics	No. of patients (n=5424) (%)	Survival (%)		*P* value^a^
		3-year	5-year	
**Age (years)**				<0.001
≤60	2739 (50.5)	71.9	61.2	
>60	2685 (49.5)	52.4	41	
**Race/Ethnicity**				0.007
White	4364 (80.5)	62.5	51	
Black	595 (11)	57.5	49.3	
Others	438 (8.0)	67.7	59.1	
Unknown	27 (0.5)	80	80	
**Gender**				<0.001
Female	2423 (44.7)	66.4	56	
Male	3001 (55.3)	59.3	47.9	
**Histology**				<0.001
Functional tumours (8150~8156)	1145 (21.1)	68	56.7	
Nonfunctional tumours (8240~8249)	4279 (78.9)	60.8	49.9	
**Location of primary tumour**				<0.001
Head	1818 (33.5)	57.6	47	
Body	746 (13.8)	64.4	54.2	
Tail	1687 (31.0)	67.9	56.1	
Duct	3 (0.1)	33.3	33.3	
Islets	135 (2.5)	74.8	60.1	
Overlapping	433 (8.0)	61.3	49.8	
NOS	502 (9.3)	55.4	46.4	
Others	100 (1.8)	74.4	69.5	
**Grade**				<0.001
Well differentiated (Ⅰ)	1888 (34.8)	81.9	73.6	
Moderately differentiated (Ⅱ)	591 (10.9)	72.8	61.3	
Poorly differentiated (Ⅲ)	366 (6.7)	31.6	24.5	
Undifferentiated (Ⅳ)	112 (2.1)	21	16.9	
Unknown	2467 (45.5)	53.9	42	
**Tumour stage**				<0.001
Localized	1681 (31)	87.4	81.4	
Regional	1212 (22.3)	73	62	
Distant	2441 (45)	42.3	29.3	
Unstaged	90 (1.7)	63.9	54.6	
**Surgery**				<0.001
Yes	3044 (56.1)	84.3	74.7	
No	2365 (43.6)	35.8	24	
Unknown	15 (0.3)	55.8	55.8	
**Regional lymph nodes**				<0.001
Positive	1153 (21.3)	74.3	62.9	
Negative	1498 (27.5)	86.3	77.9	
Unknown	2773 (51.2)	45.6	34	
**Tumour size**				<0.001
0-20mm	1226 (22.6)	78.6	71.8	
21-40mm	1806 (33.3)	63.1	52	
>40mm	2392 (44.1)	55.2	43.6	

Abbreviations: NOS=not otherwise specified. ^a^ Univariate analysis was calculated by the Kaplan-Meier method with Log-rank test,* P* value of <0.05 was considered as statistically significant.

**Table 2:** Predictors of survival identified by multivariate Cox regression analysis

Variables	Hazard ratio	95% CI	*P* value ^a^
**Age (years)**			
≤60	1.0 (reference)		
>60	1.784	1.640-1.940	<0.001
**Race/Ethnicity**			
White	1.0 (reference)		
Black	1.076	0.943-1.227	0.279
Others	0.892	0.758-1.051	0.172
**Gender**			
Female	1.0 (reference)		
Male	1.047	0.963-1.138	0.282
**Histology**			
Functional tumours (8150~8156)	1.0 (reference)		
Non-functional tumours (8240~8249)	1.0	0.907-1.103	0.998
**Location of primary tumour**			
Head	1.0 (reference)		
Body	0.893	0.783-1.028	0.117
Tail	0.918	0.827-1.019	0.108
Duct	3.271	1.045-10.239	0.042
Islets	1.051	0.81-1.363	0.708
Overlapping	0.951	0.816-1.108	0.516
**Grade**			
Well differentiated (Ⅰ)	1.0 (reference)		
Moderately differentiated (Ⅱ)	1.286	1.080-1.531	0.005
Poorly differentiated (Ⅲ)	3.040	2.581-3.582	<0.001
Undifferentiated (Ⅳ)	3.576	2.807-4.556	<0.001
**Tumour classification**			
Localized	1.0 (reference)		
Regional	2.054	1.739-2.427	<0.001
Distant	2.940	2.534-3.412	<0.001
**Surgery**			
Yes	1.0 (reference)		
No	2.713	2.354-3.127	<0.001
**Regional lymph node**			
Positive	1.0 (reference)		
Negative	0.987	0.843-1.155	0.987
**Tumour size**			
0-20mm	1.0 (reference)		
21-40mm	1.211	1.048-1.399	0.009
>40mm	1.282	1.116-1.474	<0.001

Abbreviations: CI=confidence interval. a. Multivariate analysis was calculated by the Cox proportional hazards regression model, *P* value of <0.05 was considered as statistically significant.

**Table 3:** Tumour size and survival according to disease stage

Tumour size (mm)	No. of patients (%)	3-year survival (%)	5-year survival (%)	*P* value
**All stages**	5334	62.5	51.5	<0.001
0--20mm	1205 (22.6)	78.7	71.7	
20--40	1775 (33.3)	62.9	51.6	
>40	2354 (44.1)	55.2	43.8	
**Localized disease**	1681	87.4	81.4	<0.001
0--20mm	797 (47.4)	90.8	87.1	
20--40	518 (30.8)	87.8	79.9	
>40	366 (21.8)	80.8	75	
**Regional disease**	1212	73.0	62.0	<0.001
0--20mm	173 (14.3)	84	75.9	
20--40	437 (36.1)	74.5	64.3	
>40	602 (49.7)	69.1	57.1	
**Distant disease**	2441	42.3	29.3	0.703
0--20mm	235 (9.6)	42	30.4	
20--40	820 (33.6)	42.3	28.3	
>40	1386 (56.8)	42.4	29.7	

**Table 4:** Subgroup analysis of survival by size, with or without surgical resection

Tumour size (mm)	No. of patients (5409) (%)	survival (%)		*P* value	Hazard ratio	95% CI	*P* value
		3-year	5-year				
**Surgical resection**	**3044**	**84.3**	**74.7**	**<0.001**			
0--20	906 (30)	90.5	85.6		1 (reference)		
21--40	1010 (33)	84.9	74.5		1.762	1.399-2.219	<0.001
>40	1128 (37)	79.5	68.8		2.243	1.802-2.791	<0.001
**No surgical resection**	**2365**	**35.8**	**23.4**	**<0.001**			
0--20	317 (13.4)	46.4	33.9		1 (reference)		
21--40	791 (33.4)	35.8	23.8		1.349	1.129-1.612	0.001
>40	1257 (53.2)	33.6	21.3		1.413	1.193-1.674	<0.001

**Table 5:** Demographic and clinical characteristics of PNETs≤20mm versus >20mm, SEER 1988-2013

Characteristics	Size≤20mm No. of patients (%) (n=1226)	Size>20mm No. of patients (%) (n=4198)	*P* value ^a^
**Age (years)**			
≤60	601(49.0)	2138(50.9)	
>60	625(51.0)	2060(49.1)	0.240
**Race/Ethnicity**			
White	987(80.5)	3377(80.4)	
Black	124(10.1)	471(11.2)	
Others	115(9.4)	350(8.3)	0.323
**Gender**			
Female	621(50.7)	1802(42.9)	
Male	605(49.3)	2396(57.1)	<0.001
**Histology**			
Functional tumours (8150~8156)	224(18.3)	921(21.9)	
Non-functional tumours (8240~8249)	1002(81.7)	3277(76.9)	0.006
**Location of primary tumour**			
Head	323(26.3)	1495(35.6)	
Body	244(19.9)	502(12.0)	
Tail	373(30.4)	1314(31.3)	
Others	286(23.4)	884(21.1)	<0.001
**Grade**			
Well differentiated (Ⅰ)	647(52.8)	1241(29.6)	
Moderately differentiated (Ⅱ)	104(8.4)	487(11.6)	
Poorly differentiated (Ⅲ)	32(2.6)	334(8.0)	
Undifferentiated (Ⅳ)	13(1.1)	99(2.3)	
Unknown	430(35.1)	2037(48.5)	<0.001
**Tumour classification**			
Localized	798(65.1)	884(21.1)	
Regional	173(14.1)	1039(24.8)	
Distant	235(19.2)	2206(52.5)	
Unknown	20(1.6)	69(1.6)	<0.001
**Surgery**			
Yes	906(73.9)	2138(50.9)	
No	317(25.9)	2048(48.8)	
Unknown	3(0.2)	12(0.3)	<0.001
**Regional lymph node**			
Positive	141(11.5)	1012(24.1)	
Negative	539(44.0)	959(22.8)	
Unknown	546(44.5)	2227(53.1)	<0.001
